# Durability of the Humoral Response to MPXV Infection

**DOI:** 10.1002/jia2.70165

**Published:** 2026-07-24

**Authors:** Tara N. Palmore

**Affiliations:** ^1^ Institute of Human Virology University of Maryland School of Medicine Baltimore Maryland USA

**Keywords:** antibodies, humoral immunity, mpox, vaccination

## Abstract

**Introduction:**

Mpox virus (MPXV) re‐emerged in endemic countries in recent decades, resulting in large outbreaks in central and western Africa and a worldwide epidemic of clade IIb MPXV starting in 2022. Mpox cases in vaccinated persons, and a few documented reinfections, have raised questions about the durability of MPXV humoral immunity. Longitudinal studies conducted during the clade IIb global outbreak have enriched our understanding of immune responses during and after mpox. This commentary aims to summarize current knowledge about the durability of humoral immunity against MPXV and limitations in the available evidence.

**Discussion:**

Studies following convalescent individuals show vigorous neutralizing antibody titres that peak within weeks after symptom onset and remain detectable for up to 2 years following acquisition. Binding IgG and IgA antibodies directed against MPXV antigens peak and wane more rapidly. Immunodominant epitopes, such as A35R, elicit robust responses. Older individuals who received historical replicating smallpox vaccines and acquired mpox had higher neutralizing and binding antibody titres than younger people, with no apparent difference in durability of humoral immune markers. In some studies, people living with HIV had less vigorous early antibody responses than people without HIV but without an observed difference in durability.

**Conclusions:**

Long‐term protection following mpox involves both humoral and cellular immunity. There is no identified correlate of protection for mpox. The current evidence primarily includes studies of mild to moderate clade IIb infection among cohorts largely comprising men in non‐endemic countries. Research involving additional viral clades and cohorts with greater breadth of geography, age, gender, race, ethnicity and severity of disease is needed to more fully explore durability of the MPXV immune response. A more complete understanding of this complex picture would likely inform development of improved vaccines and therapeutics for mpox.

## 1 Introduction

Mpox virus (MPXV) is a re‐emerging Orthopoxvirus that causes mpox, a disease endemic to central and west Africa. Waning population immunity to Orthopoxviruses following cessation of routine smallpox vaccination in the 1970s permitted a re‐emergence of MPXV in the decades after smallpox eradication [1, 2]. The two clades of MPXV differ in virulence in animal models and in observed human cases. Clade I is associated with more severe disease, and was recently sub‐divided into sub‐clades Ia and Ib [3]. Clade II is sub‐divided into sub‐clades IIa and IIb. MPXV clade IIb spread worldwide in 2022 in an epidemic fuelled largely by sexual contact. Globally, approximately 180,000 mpox cases have been reported to the WHO since 2022 [4], probably an underestimate of the true burden of disease. Transmission has continued, causing large outbreaks in some countries. Continued spread of multiple MPXV clades has recently resulted in detected recombination of clade IIb with clade Ib [5]. Fewer than 1% of those with documented mpox during the multi‐national epidemic of clade IIb mpox have died, with mortality associated with severe underlying immune deficiency, including advanced HIV [6]. In contrast, a 2017–2018 outbreak of clade IIa MPXV in Nigeria had a case fatality rate of 6%, also with deaths largely among people living with HIV (PWH) [7].

Mpox transmitted in the context of sexual contact has presented distinctly from mpox acquired through other routes of spread, with initial preponderance of anogenital or oropharyngeal mucosal and cutaneous disease [8]. Although smallpox was thought to confer lifelong protective immunity [9], there is uncertainty about the durability of protection after mpox. A handful of published cases of clade IIb reinfection underscore these doubts [10, 11, 12, 13], and have raised questions about whether some convalescent individuals should be vaccinated [13].

The third‐generation vaccinia virus‐based vaccine, MVA‐BN, is safe for PWH, and was used to stem the global epidemic that began in 2022. Its real‐world effectiveness has been estimated at 66%–88% [14, 15, 16, 17, 18], but infections in vaccinated persons have raised questions about the long‐term durability of protection from a replication‐deficient vaccine that elicits low titre and rapidly waning neutralizing antibodies [19, 20, 21]. Consequently, there is a lack of data regarding the need for booster vaccinations.

When the 2022 clade IIb epidemic began, durability of protection after mpox due to other viral clades was not well characterized. Researchers began to fill major gaps in understanding of immunity after mpox infection and vaccination with longitudinal clinical and immunophenotyping studies following MPXV acquisition and MVA‐BN vaccination. As a consequence, most available data on immune responses to mpox are from non‐endemic countries involving clade IIb MPXV.

## 2 Discussion

### 2.1 Immune Protection

MPXV stimulates early cellular and humoral immune responses that achieve viral clearance and induce long‐term immunity [22]. Correlates of protection are used for assessing individual host susceptibility to infection or disease severity and evaluating population immunity. No immune correlate of protection has been identified for mpox, leaving a critical gap in predicting who may experience reinfection, as well as a challenge in the development of vaccines and policies for booster vaccination after MVA‐BN. The humoral immune system has been considered fundamental to protection from MPXV based on non‐human primate challenge studies, with B cells and neutralizing antibodies established in those studies as essential to protection from severe disease [23]. However, long‐term protection from mpox, and to severe disease, likely relies on interdependent humoral and T‐cell immune components.

### 2.2 Neutralizing Antibodies

Neutralizing antibodies block viral entry and replication, and are considered a key element of immunity to Orthopoxviruses and other viral infections [23, 24, 25]. A protective threshold of neutralizing antibodies is not defined for MPXV, and was not clear for smallpox [26, 27]. Assays that measure neutralization or quantify neutralizing antibodies may correlate in vitro with protection but involve live‐virus targets requiring high‐containment laboratories and thus pose technical barriers to broader use. A range of neutralization assays and methods are used in research studies, and challenges may arise when comparing results across platforms.

In longitudinal studies of neutralizing antibodies in individuals recovering from mpox, neutralizing antibodies rise to high titres and peak 10−20 days after symptom onset, remain detectable for months [24, 25, 28, 29], and in one report for up to 2 years after infection [30], before waning.

Several studies have compared neutralizing antibodies in people recovering from mpox with those in recipients of MVA‐BN, and not unexpectedly titres are substantially higher in recently convalescent individuals [31]. Although neutralizing antibody titres are low and become undetectable within months after MVA‐BN immunization, booster vaccination provokes a strong anamnestic response. Similarly, individuals born before 1980 and presumed to have received the live replicating smallpox vaccine decades ago have rapid boosting of neutralizing antibodies following a single dose of MVA‐BN [19, 32].

### 2.3 Binding Antibodies

Assays measuring circulating binding antibodies against MPXV antigens (or their homologous VACV antigens) are more feasible and scalable than neutralization assays. The two forms of infectious virus produced in the MPXV replication cycle, intracellular mature virions (IMVs) and extracellular enveloped virions (EEVs), are functionally and antigenically distinct. IMV is implicated in early infection and transmission within the host and EEV in viral dissemination between hosts. Binding antibodies are detected against IMV antigens (A27L, A29L, E8L, H3L, M1R, A5) or EEV antigens (B6R, A35R, B2R) using a variety of assays [33].

Binding antibodies indicate immune activation and may not predict protection, based on the observation that mild disease occurred in people with documented high binding antibody titres following MVA‐BN vaccination [34]. Nonetheless, binding antibodies to key antigens may play a role in early control of infection [29]. In a notable example, clade IIb studies have shown vigorous, durable IgG responses against the EEV antigen A35R [21, 24, 29, 30]. Anti‐A35 IgG antibodies found in high concentrations in MPXV convalescent plasma have been identified as a potential therapeutic [35, 36] and advanced to pre‐clinical testing [37]. Recently, high‐affinity monoclonal antibodies against A35R isolated from convalescent humans protected mice from lethal challenge with MPXV. Patients who produced high‐affinity anti‐A35R IgG had earlier resolution of symptoms than those who did not [38].

Depending on methodology and cohort, MPXV‐specific IgA and IgG become detectable approximately 10 days after symptom onset and remain detectable for at least 1−2 years [24, 28, 39]. An ELISA validation study conducted during a 2025 clade Ib MPXV outbreak in Rwanda (in a cohort notably comprising 44% women) demonstrated IgG ratios among convalescent persons that were higher than those of recent MVA‐BN vaccinees and remained stable for at least 6 months [40].

The dynamics of mucosal immunity following mpox are incompletely understood but likely very important given the central role of mucosal inoculation and disease. In one report, antigen‐specific IgA and IgG titres against immunodominant antigens were inversely associated with MPXV viral titres in skin lesions and correlated with rapidity of viral clearance, while IgG breadth was inversely correlated with disease severity, suggesting early activation of a broad mucosal immune response to control replication [29]. In another study, circulating MPXV‐specific IgA peaked at 3 weeks and subsequently declined, with detectable IgA in less than half the cohort and mean titres below the limit of detection after 6 months [28]. Given these findings, more data are needed on functional dynamics and durability of mucosal immunity in mpox.

Memory B cells, which remain detectable for at least a year from symptom onset [21, 41], likely contribute to long‐term protection against re‐exposure and lessen severity of disease among mpox convalescent persons. Orthopoxvirus‐specific memory B cells last for decades in recipients of replicating smallpox vaccines [42], as seen in the anamnestic response in older study participants to both infection and MVA‐BN vaccination [19]. The memory B‐cell response after MVA‐BN vaccination induces a strong anamnestic response to booster vaccines as long as 5 years after primary vaccination [43].

### 2.4 T‐Cell Immunity

Durable viral‐specific T‐cell memory plays a key role in the immune response to mpox, with robust effector CD4^+^ and CD8^+^ T‐cell responses detectable early in the course of infection. Although recent research identifies a strong T‐cell response among convalescent persons, there are some important differences among their findings based on the use of different methods and vaccine comparator groups [44]. In longitudinal profiling, Wang et al. identified a durable T‐cell response with CD4^+^ and CD8^+^ T cells and T‐follicular‐helper cells, which are essential for B‐cell affinity maturation and establishment of B‐cell memory, detectable for more than a year after symptom onset [24]. The robust T‐cell response likely complements and supports the humoral immune response to generate long‐term protection.

### 2.5 Differences Between PWH and People Without HIV

In the clade IIb mpox epidemic, as with outbreaks involving other MPXV clades in endemic regions [45], clinical outcomes were more severe among those with advanced HIV and others living with significant immune compromise. The immunological basis of these divergent outcomes may not be fully reflected in recent cohort‐based immune profiling studies, as most participants had virologic suppression and CD4 counts >200 [25, 28, 29, 30, 39]. Nonetheless, some differences in humoral immune markers have emerged.

Wang et al. found no difference in neutralizing antibody dynamics among PWH and people without HIV (PWoH) [24]. In other cohorts, investigators observed differences in neutralizing and binding antibody dynamics between PWH and PWoH, but not in durability of those humoral responses [28, 39]. In one report, PWH had a less vigorous rise and more rapid waning of IgG and IgA binding antibody titres but similar durability of detection [29].

PWH and PWoH had similar T‐cell responses at diagnosis and similar proportions of MPXV‐specific CD4^+^ and CD8^+^ T‐cell responses [22] despite evidence of a less coordinated initial humoral and cellular response in PWH [29]. There were no differences in the frequency of T‐follicular‐helper cells [24].

### 2.6 Past Smallpox Vaccination

The waning population immunity to vaccinia virus that set the stage for mpox emergence is reflected in several recent studies of MPXV humoral immunity. When mpox cohorts were stratified by historical receipt of replicating smallpox vaccines—or by likely receipt inferred by birth year—some differences in antibody dynamics were noted, attributed to boosting of pre‐existing antibodies by infection. Receipt of historical smallpox vaccines, or being the age of likely receipt, was associated with higher binding antibody and neutralization titres in some studies, even at baseline, but did not necessarily extend the durability of these markers of humoral immunity [25, 29, 31].

## 3 Conclusions

Longitudinal immune profiling studies demonstrate that mpox induces an early rise of antibodies with persistent viral‐specific neutralizing antibodies, IgG and IgA, resulting in a long‐lasting humoral immune response. The humoral immune response to MPXV is accompanied by a robust cellular immune response that likely plays a critical role in protection from reinfection [24, 29]. However, protection against reinfection following mpox is not infallible, as illustrated by case reports [10, 11, 12, 13]. Infections following MVA‐BN immunization have overall substantially lower severity, with more limited lesions and fewer systemic symptoms, although vaccinated PWH who acquired mpox were less likely to be protected from severe disease than PWoH [46]. This outcome underscores the need for mpox‐specific vaccines capable of inducing strong, long‐lasting protective immunity in the populations at greatest risk.

The available evidence on durability of humoral immunity following mpox also has some substantial limitations. As noted, nearly all immune profiling studies took place during the clade IIb epidemic in non‐endemic countries, where most affected persons were male. Thus, there is a paucity of data on immune responses to other MPXV clades and immune responses in women and among individuals in endemic areas where occult Orthopoxvirus exposure may alter baseline immunity [47]. A limitation of the evidence on PWH is the disproportionate evidence from people with well‐controlled HIV when the greatest harm from mpox falls on those with substantial immune suppression. Other limitations, likely due to the ambulatory structure of most cohort studies of mpox immunity, is the weighting towards lower severity of disease. Larger cohorts with greater diversity of geography, age, gender, race and ethnicity are needed to understand durability of the MPXV immune response in the broad spectrum of humans who are affected by this disease. Data on durability of immune responses due to other clades as well as data on cross‐clade immunity are particularly needed to ensure that the next generation of vaccines and therapeutics benefit those living in the most heavily affected areas.

A further challenge in interpreting the literature on humoral immunity to MPXV is the lack of standardized serological assays for mpox. Investigators studying binding and neutralizing antibody responses to mpox and to vaccination use an assortment of assays to assess humoral immunity, and methodological differences may account for some differences in findings. This is also true of T‐cell analyses. Identifying correlates of protection for mpox is an important goal for poxvirus immunology, vaccinology and public health. Some have suggested a systems immunology approach to establish immune signatures that can predict protection [33]. Such a strategy might further elucidate duration of protection after mpox immunization and acquisition.

The crucial questions of when to administer booster doses of MVA‐BN currently await clarity from longitudinal infection surveillance, vaccine studies and immune profiling. There is not a useful serological basis for determining the timing of booster vaccination, as the decline in neutralizing and antibody titres does not apparently reflect enduring immune memory. A Congolese healthcare worker cohort receiving a booster 5 years after primary vaccination was shown to have a rapid and vigorous anamnestic response despite low baseline antibody levels [43]. Booster intervals likely need to be shorter among highly immunosuppressed persons who are at ongoing, elevated risk of exposure. There are not yet solid scientific bases on which to recommend booster vaccines in every at‐risk population. Even greater uncertainty exists around whether some persons who have recovered from mpox should be vaccinated.

In conclusion, continued investigation into MPXV immunity is vital as it continues to affect PWH and PWoH on every populated continent. Our understanding of durability and protection from reinfection may help pave the way to evidence‐based guidance for MVA‐BN boosters, development of improved vaccines that elicit more robust and durable immune responses, and medical countermeasures against mpox.

## Author Contributions

TNP authored the manuscript.

## Conflicts of Interest

The author declares no conflicts of interest.

## Data Availability

Data sharing not applicable to this article as no datasets were generated or analysed during the current study.
